# Supplementary material for: Application of Self-Assembled Raman Spectrum-Enhanced Substrate in Detection of Dissolved Furfural in Insulating Oil
Source: Nanomaterials (Basel). 2018 Dec 23;9(1):17. doi: 10.3390/nano9010017 (PMC6359157; doi:10.3390/nano9010017)
Supplement: Supplementary file 1 [file nanomaterials-09-00017-s001.pdf]

Supporting Information

Application of Self-Assembled Raman Spectrum Enhanced Substrate in Detection of Dissolved Furfural in Insulating Oil

Haiyang Shi [1,*], Weigen Chen [1], Fu Wan [1,*], Lingling Du [2], Shuhua Zhang [1], Weiran Zhou [1], Jiayi Zhang [1], Yingzhou Huang [3] and Chengzhi Zhu [4]

[1] State Key Laboratory of Power Transmission Equipment& System Security and New Technology, Chongqing University, Chongqing 400044, China
[2] Chengdu Power Supply Company, Chengdu 610041, China
[3] Soft Matter and Interdisciplinary Research Center, Chongqing University, Chongqing 400044, China
[4] State Grid Zhejiang Electric Power Company, Zhejiang 310000, China

1. The Enhancement Factor of Enhanced Substrate

The enhancement effect of SERS substrate can be evaluated by enhancement factor. There are many ways to calculate the enhancement factor, and different calculation methods have different emphasis. In this paper, the calculation formulas are as follows:

$$EF = \frac{I_{SERS} / C_{SERS}}{I_{NR} / C_{NR}}$$

where I_{SERS} is the intensity of characteristic peaks in SERS, C_{SERS} is the concentration of the sample in SERS, I_{NR} is the intensity of characteristic peaks in normal Raman and C_{NR} is the concentration of the sample in normal Raman. Under the same test conditions, the intensity of 300 mg/L furfural in transformer oil is 2541.13 in normal Raman, and the intensity of 2.5 mg/L furfural in transformer oil is 72332.06 in SERS. Therefore, the enhancement factor of substrate is 3.41×10^3.

2. The Uniformity and Repeatability of Enhanced Substrate

The homogeneity of substrates is one of the important parameters of SERS. In an experiment, 20 mg/L furfural in transformer oil was used as the target. Ten points were randomly selected on the same substrate to detect Raman signals, and the homogeneity of substrates was investigated in Figure S1. The relative standard deviation (RSD) was calculated as 8.28%, which indicated that the prepared substrate had good homogeneity.

In order to further verify the repeatability of the experiment, the consistency of substrates prepared by self-assembly on gold film surface was studied with 20 mg/L furfural in transformer oil in Figure S2. The RSD was calculated as 10.4%, which indicated that the substrate had good repeatability.

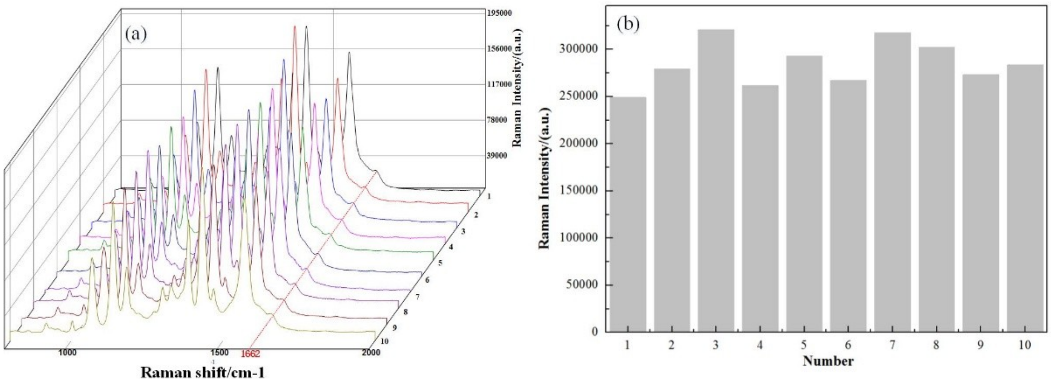

Figure S1. SERS of furfural in transformer oil at 10 acquisition points on a single substrate.

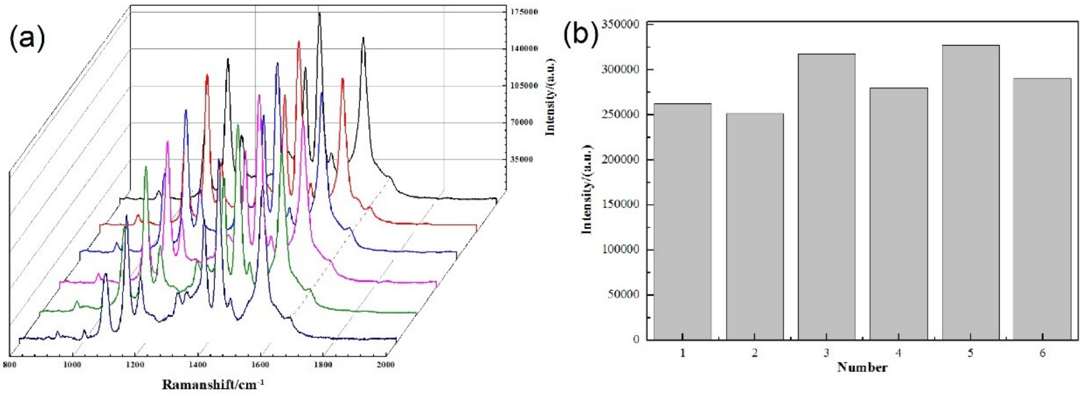

Figure S2. SERS of furfural in transformer oil on Six Substrates.
